# Angiotensin converting enzymes in patients with acute respiratory distress syndrome

**DOI:** 10.1186/2197-425X-3-S1-A91

**Published:** 2015-10-01

**Authors:** F Annoni, D Orbegozo Cortés, M Irazabal, V Fontana, FS Taccone, D De Backer, JL Vincent, J Creteur

**Affiliations:** Universite Libre de Bruxelles, Soins Intensifs, Bruxelles, Belgium

## Introduction

Angiotensin converting enzymes (ACEs) are important in the control of the cardiovascular function and their inhibition have a primary role in the treatment of hypertension and heart failure. However they have effects beyond the cardiovascular system. Angiotensin 2 production by ACE and stimulation of the angiotensin 1 (AT1) receptor subtype reduces nitric oxide bioavailability, promotes inflammation and fibrosis. The ACE type 2 (ACE2) increases angiotensin 1-7 production and counterbalances ACE effects. Some animal data have shown a beneficial role of the up-regulation of the ACE2 pathway and a detrimental role for the up-regulation of the ACE classic pathway in different ARDS, but there are no data in patients with ARDS.

## Objectives

We compared the plasma concentrations of ACE and ACE2 in patients with ARDS and correlated them with ICU mortality.

## Methods

We measured the plasma concentrations of ACE and ACE2 by a quantitative ELISA method (Cloud-Clone Corp, US) at time of diagnosis of ARDS (Berlin definition) in 80 consecutive patients admitted to our department of intensive care We compared the plasma concentrations of ACE, ACE2 and ACE/ACE2 ratio between ICU survivors and non-survivors. All analyses were performed in SPSS 22.0 and a p value < 0.05 was considered as significant. Al values are presented as medians with p25-75.

## Results

The main characteristics of the 80 consecutive ARDS patients are presented in Table 1. The ICU mortality was 31%. ACE was significantly higher in non-survivors compared to survivors, but ACE2 and ACE/ACE2 ratio were similar in survivors and non-survivors (Table 2). The area under the curve for the receiver operator characteristics (AUC_ROC) for the ACE to discriminate between survivors and non survivors was 0.65(0.51-0.79).

## Conclusions

Increased ACE levels are associated with worse outcome in ARDS patients, which might indicate endothelial activation and may have implication for therapy.
